# Mitigating childhood food insecurity during COVID-19: a qualitative study of how school districts in California’s San Joaquin Valley responded to growing needs

**DOI:** 10.1017/S1368980021003141

**Published:** 2021-07-30

**Authors:** Ashley H Jowell, Janine S Bruce, Gabriela V Escobar, Valeria M Ordonez, Christina A Hecht, Anisha I Patel

**Affiliations:** 1 Stanford University School of Medicine, Stanford, CA, USA; 2 Pediatrics, Stanford University School of Medicine, 3145 Porter Drive, F110, Palo Alto, CA94304, USA; 3 Nutrition Policy Institute, University of California, Division of Agriculture and Natural Resources, Oakland, CA, USA

**Keywords:** COVID-19, Food insecurity, School meals, Childhood nutrition, Latino

## Abstract

**Objectives:**

To explore best practices and challenges in providing school meals during COVID-19 in a low-income, predominantly Latino, urban–rural region.

**Design:**

Semi-structured interviews with school district stakeholders and focus groups with parents were conducted to explore school meal provision during COVID-19 from June to August 2020. Data were coded and themes were identified to guide analysis. Community organisations were involved in all aspects of study design, recruitment, data collection and analysis.

**Setting:**

Six school districts in California’s San Joaquin Valley.

**Participants:**

School district stakeholders (*n* 11) included food service directors, school superintendents and community partners (e.g. funders, food cooperative). Focus groups (*n* 6) were comprised of parents (*n* 29) of children participating in school meal programmes.

**Results:**

COVID-19-related challenges for districts included developing safe meal distribution systems, boosting low participation, covering COVID-19-related costs and staying informed of policy changes. Barriers for families included transportation difficulties, safety concerns and a lack of fresh foods. Innovative strategies to address obstacles included pandemic-electronic benefits transfer (EBT), bus-stop delivery, community pick-up locations, batched meals and leveraging partner resources.

**Conclusions:**

A focus on fresher, more appealing meals and greater communication between school officials and parents could boost participation. Districts that leveraged external partnerships were better equipped to provide meals during pandemic conditions. In addition, policies increasing access to fresh foods and capitalising on United States Department of Agriculture waivers could boost school meal participation. Finally, partnering with community organisations and acting upon parent feedback could improve school meal systems, and in combination with pandemic-EBT, address childhood food insecurity.

COVID-19 has led to an unprecedented social and economic crisis. Certain populations, including low-income communities, racial/ethnic minorities, immigrants and those with a lower education status, have faced a disproportionate burden of the negative impacts of COVID-19^(1,2)^. Rising unemployment has heightened food insecurity defined as reduced access to affordable and nutritious food^(3)^. This is important as food insecurity is a major determinant of poor dietary quality and adverse health outcomes (diabetes^(4)^, hypertension^(5)^, CVD^(6)^, depression^(7)^, overall morbidity^(8)^). Households with children are particularly at risk, leading children in these families to suffer lifelong adverse health and learning consequences^(3,9)^.

Although the Supplemental Nutrition Assistance Program is the nation’s largest and most important nutrition assistance programme in the country’s safety net, the child nutrition programmes administered by the United States Department of Agriculture (USDA) also play an essential role in addressing food insecurity. Through the National School Lunch Program, School Breakfast Program, and Child and Adult Care Food Program, millions of children receive breakfast and lunch in schools, and snacks and supper in afterschool programmes^(10)^. Even during summer, children can receive meals through the Summer Food Service Program or Seamless Summer Option^(10)^. School meals, which must meet federal nutrition standards, are generally healthier than meals from home, particularly for low-income populations and also can improve academic performance^(11,12)^. Participation in child nutrition programmes can also help reduce food insecurity and obesity^(13,14)^.

Beginning in March 2020, US schools closed in-person instruction to stem COVID-19 transmission. Although the USDA did not require schools to serve meals during closures, many school districts rapidly responded to serve families through innovative measures^(11)^. In March 2020, Congress passed the Families First Coronavirus Response Act (H.R. 6201 Public Law: 116–127) which allowed states to apply for waivers granting flexibilities in implementing child nutrition programmes. For example, waivers permitted pick-up of multiple or several days of meals in batches (Waiver #1), allowed school meals to be served ‘off-site’ in non-congregate settings (Waiver #2), allowed parents to pick up school meals without children present (Waiver #5), permitted flexibilities in nutrition standards – normally all of which are required for federal meal reimbursement (Waiver #13) and provided area-wide eligibility so all children were eligible for free meals (Waiver #14). To offset reduced access to school meals, the Act also allowed states to apply for the pandemic electronic benefits transfer (P-EBT) programme which provided monies to families of all children who were eligible^(15,16)^.

A recent US study, which analysed different strategies that jurisdictions implemented to guide districts in distributing and providing information about school meals, found that although many states offered implementation guidance to districts, more tailored information was needed. Specifically, this study found that more locally applicable information, for example, regarding communication strategies with families about school meals or details about partnerships with anti-hunger organisations, could provide additional support to food service directors serving children vulnerable to food insecurity^(17)^.

Despite these USDA waivers and outreach efforts, participation in school meals has decreased since the pandemic. In the first 9 months of COVID-19, school meal programmes served 30 % fewer students, resulting in an over $2 billion revenue loss for child nutrition programmes. This not only represents a missed opportunity for reducing food insecurity but also adversely impacts districts’ finances^(18,19)^.

Few studies explore how school districts have modified meal programmes during COVID-19^(20)^. One study examined online district information to describe innovations in rural districts^(21)^. A second study described case studies of strategies four urban US districts used to increase school meal access during school closures^(22)^. Most of the existing research has focused on food service directors, who play a major role in determining how meals are served to children. Yet, other stakeholders are also involved in influencing meal operations, including the school district board, school superintendent, parents and community partners. Furthermore, although schools must meet national and state requirements as outlined by the USDA, individual districts do have some authority in their implementation of meal programmes^(15)^.

To our knowledge, no study has investigated varied perspectives of school district officials, parents and partner organisations regarding school meal provision during the pandemic, and particularly in a predominantly low-income, Latino immigrant community. Gaining a rich understanding of school meal challenges and best practices in under-resourced communities from multiple perspectives can inform school meal programming and policies amidst COVID-19, and be used to build a more resilient school meal system that can better serve families in low-resource settings in the future^(23)^.

## Methods

### Community academic partnership

This study leverages a community–academic partnership among two non-profit community organisations (Cultiva la Salud and Dolores Huerta Foundation) working towards health equity and social justice in the San Joaquin Valley (SJV) of California and two academic institutions (Stanford University and Nutrition Policy Institute). This partnership is rooted in years of collaborative research between community-based organisations and researchers on this team. This research used a ‘community-based participatory research’ approach, where academic institutions and community partners collaborated throughout the research process. Community partners drove the study objectives, selected participating study districts, identified relevant questions, shaped methods, determined recruitment practices and engaged in data collection with parents^(24)^. To ensure that all partners were able to engage equitably in the research and have their voices prioritised, community–academic partners met weekly to confirm priorities, review project updates, solicit feedback and discuss next steps. Finally, the preliminary study findings were shared throughout the research process with community partners so that results could inform their advocacy and on the ground efforts as quickly as possible to address social inequities. As an example, the partners are currently using an infographic of study findings to advocate for changes to improve school meal participation in study school districts.

### Study design and community context

This study focuses on SJV, a region with a prominent agricultural and food processing industry that is home to many farmworkers and a majority Latino population^(25,26)^. The region experiences some of the highest rates of food insecurity and diet-related chronic diseases in California. Pre-COVID 2018 rates of childhood food insecurity ranged from 18 to 24 % in SJV counties compared with 15 % statewide, and 46 % of adults living in SJV experience at least one chronic disease, compared with 41 % across California^(25,27,28)^. SJV is also a hotspot for COVID-19 cases and mortality^(29)^.

In this qualitative study, we conducted semi-structured interviews, which are interviews that included both open-ended and closed-ended questions, with stakeholders including school officials (e.g. food service directors, superintendents) and partner organisations, and focus groups with parents from six SJV school districts^(30)^. Our goal was to understand multi-stakeholder perspectives of best practices and challenges in implementing school-based meal programmes during COVID-19 in an under-resourced, predominantly Latino, urban–rural region.

### Study sample and recruitment

The community–academic partnership selected six school districts across the SJV region to participate. Districts were prioritised based on partners’ existing relationships with families and school officials as well as geographic diversity^(31)^. The six SJV school districts participating in the study were located in different geographical settings: town (*n* 4), city (*n* 1) and rural (*n* 1). Student enrollment ranged from 600 to 73 000 students (median=4873, sd = 28 226·7). Most districts served predominantly Latino (median 93 %, sd = 11 %) and low-income students receiving free- and reduced-price meals (median 92 %, sd = 7 %).

For semi-structured interviews, we first emailed district food service directors and superintendents requesting an interview. We followed up via email or phone call as needed. Using snowball sampling, we asked food service directors and superintendents to identify additional key school meal stakeholders^(32)^. To understand parents’ experiences, we conducted focus groups in English and Spanish with parents across the same six districts who community partners recruited via social media and phone calls.

### Data collection

Interviews and focus groups occurred from June to August 2020. The lead author (A.H.J.) conducted eleven 30–60 min key stakeholder interviews via Zoom. In collaboration with community partners, researchers (V.M.O., G.V.E., and A.H.J.) conducted six 60-min focus groups with parents via Zoom (5 = Spanish; 1 = English). All facilitators underwent training to ensure focus groups were standardised^(33)^. Key stakeholders included: food service directors (*n* 5), superintendents (*n* 2) and partner organisations (*n* 4) (e.g. funders, food cooperative). Parent participants (*n* 29) were 100 % Latino and majority Spanish speaking (79 %) (Table 1).

**Table 1:** Study participants by geographical setting, San Joaquin Valley, June–August 2020

Overall participants	Total *n* 40		Rural *n* 7		Town *n* 26		City *n* 7	
	*n*	%	*n*	%	*n*	%	*n*	%
Food service directors	5	12	1	14	3	12	1	14
School superintendents	2	5	1	14	1	4	–	
Partner organisations	4	10	1	14	2	8	1	14
Parents (total)	29	73	4	57	20	77	5	71
Spanish speaking			4		14		5	
English speaking			–		6		–	

The community–academic partnership collaboratively developed the semi-structured interview and focus group guides. Question domains included: service during non-instructional periods; strategic community partnerships; children’s and families’ needs; P-EBT; future plans; and lessons learned (online Supplementary Table).

School meal participation was assessed qualitatively based on interviews with food service directors. All food service directors are required to track school meal participation, which is submitted to the USDA for reimbursement. Food service directors interviewed understood how their meal participation had changed during the study period as compared with previous years.

All participants were offered a gift card for their time (stakeholders received $25-value; parents received $50-value). The grants listed in the acknowledgements funded these incentives and supported some effort for investigators and community partners to work on the study. Verbal consent was obtained prior to participation. Stanford University’s Institutional Review Board approved this study.

### Data organisation and analysis

Interviews and focus groups were audio-recorded and transcribed verbatim and inputted into Dedoose, a qualitative software that enables researchers to share transcripts collaboratively, code data, assess interrater reliability and organise data for theme analysis^(34)^. Three primary coders led codebook development with feedback from team members and community partners^(35)^. After independently coding a subset of transcripts, primary coders met regularly to discuss coding processes and discrepancies^(30)^. The final codebook had a pooled Cohen’s *κ* score ranging from 0·78 to 0·91 between coders^(36)^.

Following coding, primary coders identified themes that were extensively discussed and modified with team input^(37)^. Themes were shared with community partners for feedback^(38)^. Subsequently, our community–academic team disseminated findings through two webinars for school meal stakeholders and parents. Webinars allowed participants to learn best practices and enabled validation of findings^(39)^.

The social–ecological model provided a framework that guided our research methods and analyses. This model identifies five levels that impact health: intrapersonal, interpersonal, institutional, community and public policy^(40)^. We incorporated this model in the development of interview and focus group questions, and in our sampling of participants from the school, parent, community and administrative levels, as they all represent key levels of influence (online Supplementary Table). We also used the social–ecological model as a framework for our analysis to organise multi-level themes impacting school meal service during COVID-19.

## Results

**Fig. 1:**
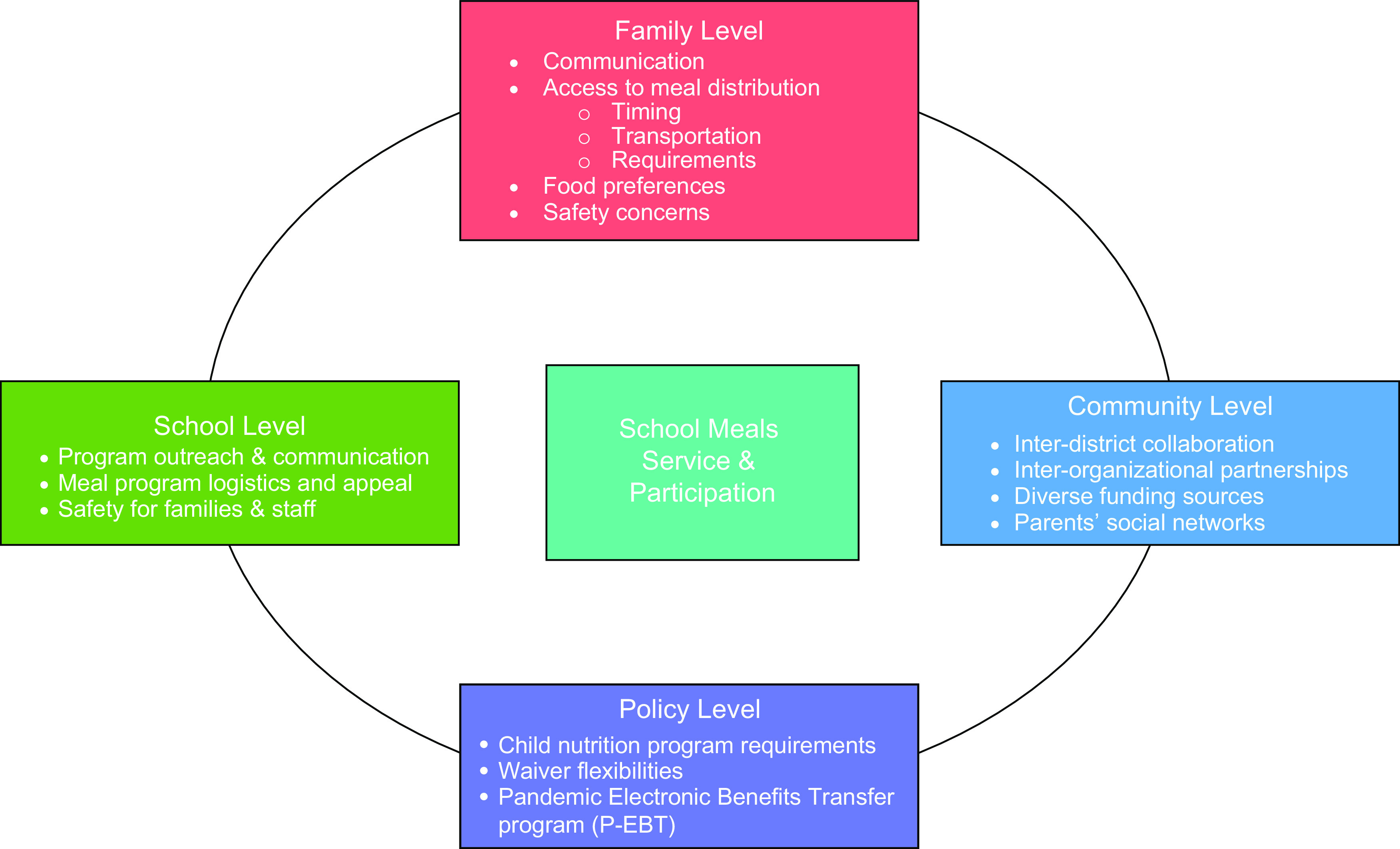
Adaptation of the social–ecological model based on theme analysis to school meal service and participation during the COVID-19 pandemic, San Joaquin Valley, June–August 2020

Our results are organised below based on the multi-level themes derived from the social–ecological model that impact school meal service and participation during COVID-19: school (community level), family (intra- and inter-personal level), community (community level) and policy (policy level) (Fig. 1)^(40)^. Qualitative themes are summarised in Table 2.

**Table 2:** Considerations for providing school meals during the COVID-19 pandemic by social–ecological level, San Joaquin Valley, June–August 2020

Framework	Key programme considerations	Exemplary quotes
Family and school level	Programme outreach • Social media: Multiple strategies used to communicate simple and concise information regarding school meals to parents (e.g. news outlets, social networks and community group chats) • Direct outreach to parents: Direct parent contact through emails, text messages, flyers, mail and pre-recorded Robo phone calls • Interpersonal communication: Many parents learned about school meals through word-of-mouth outreach with personal contacts, community members, school families, teacher announcements and trusted community organisations Meal programme logistics • Pre-packaged meals: Pre-packaged meals allowed efficient and safe meal distribution • Multiple pick-up options: Grab-and-go meals, bus stop pick-up, multiple sites aimed to improve meal accessibility • Shifted hours: Shifted meal service hours to earlier in the day to reduce exposure to the heat • Batched meals: Batched (fresh or frozen) meals used to minimise the number of times parents had to travel to access food Meal appeal • Temperature concerns: High ambient temperatures impacted food quality • Healthy, child-friendly food: Parents desired healthy and fresh food for their children that was child-friendly and varied COVID-19 safety concerns • COVID-19 safety trainings: Some districts reinstituted training for all available staff to ensure proper food handling and social distancing • Trained personnel only: Only trained staff able to participate in food distribution to enforce food safety and minimise COVID-19 spread • Contactless food distribution: Some schools deposited grab-and-go meals directly into open trunks to minimise exposure; bus stop delivery also enforced social distancing protocols	*‘We serve from four different sites in our community over the summer. And so, we have kind of community hubs where folks can come and pickup meals from four different sites in the city. Our city is not that large, but we tried to designate locations that would be easy for folks to navigate… located in different quadrants of the city’. - School Superintendent* *‘[I picked up] the same food for four days [in a row]. So, I stopped going. I really enjoyed the fresh fruit and vegetables, but not the canned food. I thought they were getting too much bready food and it was not healthy. I really liked the fresh options’. – Parent* *‘There are some regions with high numbers of families working in the fields that don’t have as great access to technology that we would pass on the flyers and information. But we did a lot of work on social media so that made me think are we doing enough for families who are not as active on the internet or only have calls or texts’ - Food Service Director*
Community level	Partnerships • Inter-district collaboration: Some districts communicated with one another through a cooperative and/or regular calls that enabled them to share tips, best practices and food options • Inter-organisational partnerships: Distribution of supplemental resources beyond school meals (e.g. food pantry items, diapers, formula, masks and cooling centre information) • Funding: Grants to support school meals during COVID-19 provided by external organisations were critical for school districts (e.g. funding for additional staff salaries, gas for food delivery vans, cooling fans or packaging materials) • Fresh foods: Some districts strived to increase families’ access to fresh produce, such as by purchasing food through the Department of Defense Fresh Fruits and Vegetables Program, or applying for grants from partners	*‘The food pantry… gave a lot of things like canned foods or dry cereals and beans… [which] gave me more to be able to feed our whole family’. –Parent* *‘Our nutrition director… went for a fresh fruit grant and she was given $25 000 and we bought the most beautiful fruit [and] produce…, we were sending home fresh blueberries, oranges, strawberries, avocadoes, and it was amazing, what these kids were getting in their meals’. – School Superintendent*
Policy level	• Child nutrition programme waiver flexibilities: Allowed area-wide eligibility so that in low-income communities all children might receive free meals, removed child requirement to eat meals on site, allowed for parent pick-up of single or batched meals, enabled flexibility in serving time • Pandemic-electronic benefit transfer (P-EBT): Parents highlighted their appreciation for P-EBT that allowed them to purchase items for home cooked meals; districts echoed the utility of this resource for families	*‘COVID definitely …terrifies our families. So we were very fortunate when the USDA approved for parents to come pick-up meals without having their children in the vehicle with them or walking to the site and not having their children with them’. – Food Service Director* *‘The best [resource] was the P-EBT cards. [The P-EBT cards] allowed me to get groceries for my children. I could go on my own time and not worry about whether or not I had food available’ – Parent*

### School- and family-level factors impacting meal service

#### Programme outreach to improve meal participation

School food service directors described communicating with families about meal distribution times, pick-up locations and options through programme outreach (e.g. flyers, signs, emails, texts, robo-calls). Most directors commented that frequent communication in English and Spanish across diverse platforms was necessary for strong participation. Communication methods included social media and website updates, calls, texts, flyers distributed across community sites (e.g. grocery stores, doctors’ offices) and outreach to parent groups. In the words of one food service director:

‘We promote our program, and we go out to the community. We pass out flyers, we post on our website, and we post on social media…yesterday was a very slow day… so today we are out in the community again and we are passing out those flyers.’

Parents voiced preferences for frequent, low-barrier communication (e.g. texts, flyers) they could easily reference later. Some parents described how English-only or internet-focused communications limited their access to information. They explained that insufficient communication about changes in distribution logistics (e.g. hours, locations) prevented some families from accessing meals, and added that parents often relied on updates from other parents.

#### Meal programme logistics and appeal

As understandings of COVID-19 transmission improved, school districts needed to adapt rapidly to changing safety guidelines while balancing needs of students, families and staff. Food service directors commented how difficult it was at the pandemic’s onset to disrupt their food service processes and design new COVID-19 safe meals, particularly as many commodities for menus require pre-ordering. While all study districts provided meals despite COVID-19 school closures, their strategies, and extent of service, varied. A handful of districts that served meals during spring 2020 distance learning suspended summer meal operations due to low participation.

All food service directors chose to implement grab-and-go meals, which parents overwhelmingly appreciated. Food service directors offered morning and/or lunch pick-up, but most distributed morning meals to avoid SJV heat. Parental work schedules and children’s virtual learning made morning meal pick-ups difficult for some families. As one parent described:

‘The lunchtime pickup was too early. I am at work from 6am-3pm so it was hard to get lunch for my kids. I hope they [school officials] change that because if they continue to do this, I will not be able to attend since I will be at work or I will be commuting because my work is far from home.’

In particular, agricultural labourers working dawn to dusk desired late-afternoon meal distribution.

Transportation barriers exacerbated parents’ meal pick-up challenges especially if parents lacked a car or distribution sites were distant. As detailed by a parent:

‘Many families who live on the outskirts of the district were not supplied well with food because they had to drive so far to get them [meals] at the designated pick-up spots. It was also hard for families who did not have cars and so many stayed without lunch during this time.’

Districts implemented diverse strategies to address accessibility. A majority of districts served meals in ‘batches’, providing multiple meals or days of food at once. This reduced the number of trips parents made to pick up meals, which they appreciated. One district also included weekend-batched meals. Some districts delivered meals to locations in closer proximity to families including apartments, parking lots and bus stops to which children could walk and pick up their own meals. Some parents stated that multiple grab-and-go sites improved food access.

While many districts allowed families to pick up meals without children present, not all districts took advantage of that waiver. In such districts, parents faced additional challenges including mobilising large families and disrupting virtual learning. In districts allowing child-free pick-up, some parents worried about leaving kids alone. A few districts allowed parents to pick up meals for multiple families, which reduced meal access burdens.

Many food service directors needed to provide pre-packaged and frozen grab-and-go meals to satisfy food and pandemic safety guidelines. Nonetheless, some worked to serve children fresh options. A food service director described their collaboration with a regional cooperative to secure unused Department of Defense Fresh Fruits and Vegetable Program funding to boost produce offerings.

Most parents expressed a desire for child-friendly and healthy meals with fresh produce that they believed would improve their children’s health. However, there were differing opinions among parents about school meal quality, even within the same district. While some parents emphasised that their children liked school meals, especially the supplementary dinners and fresh fruits provided, many other parents perceived packaged meals as ‘unhealthy,’ ‘not nutritious’ and ‘monotonous.’ Some parents complained food was ‘rotten’ and ‘soggy’. Parents in households dissatisfied with meal quality were less willing to overcome barriers to access food. In the words of one parent:

‘I did stop picking up lunches for a little while because it just did not seem like it had any nutritional value for [my son]. So instead of going out of my way to pick up the lunches, I would just cook something at home because I didn’t need to have any more corn dogs or I didn’t know if they [school officials] were going to actually give them something different.’

Finally, some parents mentioned limited opportunities to provide the district with feedback, which left them feeling disempowered and unheard.

#### COVID-19 safety concerns

Food service directors prioritised staff and family safety, as many districts were located in the ‘hardest hit COVID-19 counties’. Safety measures included contactless grab-and-go packaged meals, staff training on safety precautions and utilisation of personal protective equipment to reduce risk of COVID-19 transmission. Many districts described difficulty guaranteeing a reliable source of funding for supplies needed to implement safety protocols (e.g. personal protective equipment, food packaging materials), thus limiting meal provision.

Parents conveyed concern about COVID-19 transmission when leaving cars to pick up meals at grab-and-go sites. As one parent described, ‘One [reason why it’s hard] is due to the issue of the contagion…and at the beginning they were not taking good precautions and I was afraid about putting my kids at risk’. Other safety concerns included those of leaving their children unsupervised at home or waiting alone in the car. Another parent emphasised, ‘When I went in the beginning [to pick up meals] it was difficult because I had a baby. It was dangerous for me to take them in the car and so sometimes I chose not to go at all because I had no one to leave them with. My oldest is 11 and leaving them alone is not a safe option’. ‘Long lines’ and ‘congested’ locations were particularly frustrating, and parents appreciated schools that implemented contactless pick-up and enforced social distancing. Finally, a handful of parents preferred picking up meals at school cafeterias rather than sending children to bus stop pick-up locations, as they perceived it was challenging for children to remain socially distant in lines.

### Community-level factors impacting meal service

#### Inter-district collaboration

Food service stakeholders highlighted inter-district collaboration that helped them serve school meals effectively during COVID-19. Two food service directors commented on their partnership with a regional cooperative that manages member district food commodities. This cooperative increased collaboration between districts to share resources and trade commodity food and produce. Another food service director gleaned best practices by participating in phone calls with food service stakeholders where they received waiver updates and leadership tips.

#### Inter-organisational partnerships

Districts engaged in collaborative partnerships with community organisations including food banks and public works departments. These partnerships provided additional resources (e.g. food pantry items, diapers, masks, cooling centre information) during school meal distribution. They were mutually beneficial for districts and partner organisations as they boosted school meal participation and public appreciation of local services. As one food service director described, ‘We partnered with food banks…and last week they brought 500 boxes of food items…to distribute at the sites…that went very well, we had parents that were very appreciative’.

#### Diverse funding sources

Low participation in school nutrition programmes led to decreased reimbursement from USDA forcing some school districts to discontinue summer meal service and threatening closure for many others. Financial deficits from lower participation were exacerbated by costs of providing meals during a pandemic including expenses for packaging meals for safety and transport (e.g. bags for ‘grab-and-go’ meals), increased staff salaries for longer working hours and in hazardous conditions, transportation to deliver meals, cooling fans for staff to provide food outdoors rather than indoors and funding for staff personal protective equipment.

School districts sought diverse funding sources to address these financial challenges. Many districts jointly received funding from a national organisation that provided discretionary funds to meet additional needs associated with serving meals during COVID-19, and some food service staff voiced that this funding was essential to continuing meal operations. Interviews with funders highlighted how their organisations shifted to ‘providing grants to assist schools in [COVID-19] feeding efforts’. Other school districts highlighted the importance of supplemental federal governmental funds to cover costs. However, sometimes school districts would have to dip into their savings to fund meal provision as they waited for the federal emergency reimbursement. When participation in school meals was low, some food service directors responded by increasing community outreach to boost participation; this in turn helped to feed more families and helped to cover some COVID-19-related costs.

### Policy-level factors impacting meal service

#### Child nutrition programme waiver flexibilities

In the 2010 Healthy, Hunger-Free Kids Act, Congress required that federal child nutrition programme standards align with the current Dietary Guidelines for Americans, and USDA has developed and implemented these improved standards. Other USDA requirements address concerns such as programme accountability and safety. However, during COVID-19, districts utilised waiver flexibilities allowing them to operate school meal programmes under trying pandemic conditions. Food service directors frequently noted the critical nature of non-congregate meals, which allowed children to eat outside of the usual cafeteria setting (Waiver #2)^(15)^. School personnel also highlighted the importance of serving free meals to all children in their community, utilising ‘area eligibility’ (Waiver #14)^(15)^. Directors appreciated flexibilities allowing them to serve at different hours to meet community needs and avoid heat (Waiver #1)^(15)^ and some food service directors noted the usefulness of the waiver allowing parents to pick up meals without children (Waiver #5)^(15)^. Some commented on the challenges of keeping up with frequent waiver changes that inhibited adequate planning. A superintendent desired relaxed federal guidelines and more local control of funds to enable rapid response in emergencies.

Parents appreciated the ability to pick up meals without needing to bring children (Waiver #5)^(15)^. Other flexibilities parents praised included the ability to pick up all meals for their children at one distribution site or school (Waiver #14)^(15)^ and non-congregate feeding that allowed children to eat ‘grab-and-go’ meals at home to minimise risk of COVID-19 transmission (Waiver #2)^(15)^.

#### Pandemic electronic benefits transfer

Among parents, P-EBT was a highly appreciated and beneficial resource during COVID-19. As one parent described, ‘with three kids we were scared but once [we got the P-EBT] it really helped a lot to feel better about being able to provide for them’. While some parents faced P-EBT application and activation challenges, such as information in English only, all parents liked the freedom of choice in spending their P-EBT benefits at the grocery store, buying the foods their family enjoyed and the elimination of logistical barriers to access meals from school that P-EBT benefits offered. Parents uniformly desired P-EBT programme renewal in the academic year.

While some food service directors perceived that P-EBT decreased school meal participation, overall they emphasised its important benefits to mitigate food insecurity. The majority of study districts provided application information for P-EBT.

### Future plans

School district stakeholders described strategies for serving future meals depending on diverse school re-opening scenarios. Most districts engaged in discussions to develop re-opening plans addressing student, parent and staff needs. District stakeholders expressed the need for clarity on the status of waiver extensions to allow them to prepare and serve their community. Parents were specific about changes they wished to see, including more fresh produce in school meals, flexible meal pick-up times at locations throughout their community and a meal delivery system.

## Discussion

To our knowledge, this is the first study to investigate the unique challenges to school meal programmes in urban–rural, under-resourced and largely Latino communities during a pandemic. Our study found that efforts to make meals accessible were not enough to overcome factors such as meal appeal, ultimately reducing participation in many districts. Without adequate participation, school meal programmes are neither financially stable nor fully addressing increasing rates of food insecurity. We summarise recommendations for increased participation in Table 3.

**Table 3:** Recommendations to increase participation in child nutrition programs

Agents for change	Rationale for action based on study findings	Child nutrition programmes recommendation for action
School district, State education department, USDA FNS*	Parents desire means to provide input on children’s school nutrition as well as up to date meal programme information	Develop modalities (e.g. meetings, wellness policy, written communication) to increase bi-directional home-to-school and school-to-home communication
School district, State education department, USDA FNS	Parents want school meal communication in their own language that they can access and easily refer to later	Prioritise communications that are culturally, linguistically and technologically accessible
School district, State education department, USDA FNS	Parents desire fresh food for their children; co-benefits include healthier dietary intake and support of local economies	Develop mechanisms to support local sourcing by identifying certain school districts to serve as food ‘hubs’; hubs would serve as procurement centres with enhanced infrastructure and expertise
USDA FNS	Build on the success of COVID-19 waiver flexibilities to provide a seamless transition when emergencies demand it	Institutionalise emergency accessibility operations for school meals

* United States Department of Agriculture Food and Nutrition Service.

Districts worked to address logistical and safety concerns inherent to serving school meals remotely. At the start of COVID-19, district staff had to rapidly adjust menus and reconfigure their well-oiled meal distribution processes. Districts faced ongoing challenges including maintaining safe operations, ensuring staff had time away from work, navigating waiver issuance, boosting low participation and funding pandemic-related expenses. Existing literature on COVID-19 school meal operations in other communities and states similarly reports lower participation, difficulty addressing safety concerns and varying uptake of waiver flexibilities across districts^(15,22)^. Districts in SJV utilised strategies similar to those in the literature to respond to challenges, including batched meals, pick-up locations throughout the community, bus-stop delivery systems and multilingual meal outreach^(15,20,22)^.

Our investigation found that study districts thought they were serving meals well despite the myriad of challenges. However, parents often felt differently, citing poor communication about logistics, the lack of information in Spanish and transportation difficulties as barriers to accessing meals. It is critical that school meal information and outreach be culturally and linguistically relevant for all families, including those that have limited digital literacy. Additionally, parents wanted fresher and more child-friendly food that was varied and included fruit or healthy snacks such as yogurt. They frequently mentioned concerns about school meals that appeared unappealing, unhealthy and monotonous, which disincentivised participation. Pre-pandemic studies of low-income communities have similarly found that parent perceptions of school meal healthfulness are associated with child participation^(41,42)^. It is tragic that families living in the nation’s top producing state for fruits and vegetables noted lacking access to nutritious and fresh foods, and our findings suggest efforts to increase freshness and appeal could improve school meal participation. We hypothesise that pre-packaged frozen and processed meals may bias parents to perceive meals as unhealthy, and that developing more appealing packaging could increase participation. Further, we speculate that parents were not sufficiently informed that school meals met nutritional standards, resulting in a potential disconnect between parents’ perception of and actual nutritional quality. Improved communication between parents, community non-profit organisations and districts about school meal nutrition standards together with a means for parents to provide feedback on school meals could boost participation.

Our study found that collaborative partnerships helped many districts overcome challenges and meet families’ needs while simultaneously enhancing outreach and marketing for community organisations. The literature on COVID-19 school meal programming echoes the importance of cross-sector collaboration and local partnerships to mitigate food insecurity^(15,22)^. Future partnerships could leverage the fresh produce grown in SJV to provide families with healthy and culturally appropriate foods to increase school meal participation.

Our findings demonstrate the important role of novel federal nutrition policies in ensuring school meals were provided during COVID-19. Without USDA waiver flexibilities, critical innovations including grab-and-go meals and parent pick up of meals without children present would have been impossible^(15)^. While the option to relax nutrition standards may have enabled districts to serve a greater variety of pre-packaged options (Waiver #14), data on the impact of these leniencies on children’s diets are lacking. Parents were also overwhelmingly positive about the newly instituted P-EBT programme as it gave them autonomy and allowed them to avoid barriers to accessing school meals. This sentiment was validated by California’s high P-EBT participation with 95 % of eligible families participating in the programme statewide^(16)^. While some may worry about the nutritional value of foods parents purchase with P-EBT, study parents noted buying more nutritious items than those offered at school. Thanks to advocacy efforts, P-EBT was extended to September 2021^(43)^.

Our findings also suggest complementary benefits of P-EBT and school meals. While P-EBT can help mitigate difficulties accessing school meals, it may be challenging for some busy families or homeless and foster youth to have time or means to purchase and prepare low cost, healthy food. Moreover, rural areas may have limited access to grocery stores, farmers markets and healthy food providers, and urban areas may be oversaturated with unhealthy options^(21,44)^. Given such constraints, schools meals and P-EBT are both needed, particularly now when rates of food insecurity have skyrocketed^(15)^.

Study limitations include the size, lack of generalisability and our inability to gather perspectives from every key stakeholder. Additionally, although study communities include a variety of ethnic minorities, focus groups included only Latino parents since our community partners, who work primarily with Latino families, recruited participants. Study strengths include the ‘360°’ perspectives obtained, its timeliness and its contribution to the limited literature on strategies to address food insecurity in low-resource agricultural communities. Future research should include greater parent diversity, explore school meal innovations and challenges into the school year and investigate characteristics allowing some districts to serve superior school meals.

## Conclusions

This paper has important public health and nutrition policy implications. Policies and programmes that increase access to fresh and appealing foods and improve home-to-school communications about the meal programme could boost participation in school meals during COVID-19 and beyond. The success of P-EBT suggests its ongoing value both during the COVID-19-related economic downturn and as a supplement to the Summer Food Service Program that typically has lower participation. Finally, leveraging external resources, partnering with community organisations and developing systems for eliciting and acting upon parent feedback regarding nutrition programmes could lead to a more resilient school meal system that can reduce food insecurity and improve child health and well-being.

## Supplementary material

For supplementary material accompanying this paper visit http://dx.doi.org/10.1017/S1368980021003141. click here to view supplementary material
