# Supplementary material for: Mitigating childhood food insecurity during COVID-19: a qualitative study of how school districts in California’s San Joaquin Valley responded to growing needs
Source: Public Health Nutr. 2021 Jul 30;26(5):1063–73. doi: 10.1017/S1368980021003141 (PMC8367873; doi:10.1017/S1368980021003141)
Supplement: Supplementary file 1 [file S1368980021003141sup001.docx]

**Supplementary Table 1: Sample Interview Questions for Semi-Structured Interviews and Focus Groups with Key Stakeholders and Parents regarding School Meal Service and Participation, San Joaquin Valley, California, June-August 2020**

| **Question Purpose** | **Questions for school district staff (food service directors and superintendents)** | **Questions for Parents** | **Questions for Partner Organizations** |
| --- | --- | --- | --- |
| **Service during non-instructional periods** | - Some schools are finding it hard to serve meals all summer long. Can you tell me how things are going, and any challenges you have faced?   - *If not planning to serve meals all summer*: What would you need to provide meals the entire summer?   - How do you think participation rates are going to look / do look during summer? - We understand in the San Joaquin Valley it’s very hot during the day. What have you done to avoid spoilage? | - What has made it easy to get meals from school while schools are closed for COVID-19? - What has made it hard to get meals from school while schools are closed for COVID-19? - How did you find out about school meals in your community? - Which type of communication was most helpful to learn about school meal updates? | - Your school district has/has not been able to serve recently. How has this impacted your community/ programming? |
| **Strategic community partnerships** | - Can you please describe any important partnerships that you developed with external organizations (e.g., local food banks, libraries, community organizations)   - How did these partnerships support you with summer meal outreach?   - How, if at all, did these partnerships help to boost participation in your programming? | - Can you please describe any other resources or services that you received when accessing school meals? (e.g., diapers, books) | - Can you please describe your partnership with the school district to support school meals? |
| **Children and Families’ Needs** | - How did your program assess and respond to the needs of children and families during the pandemic? | - Tell me your experience with the school meals - how did your school district do in responding to the food needs of your family? - Sometimes schools aren’t serving meals during breaks - what have you all been doing to supplement during the times when schools aren’t serving? (e.g., pantry food, to go meals, hot meals) | - How did your organization assess and respond to the needs of children and families? |
| **Pandemic EBT** | - How has Pandemic EBT affected your program? - What has been your role in informing parents about Pandemic EBT? | - Did you receive Pandemic EBT cards for your children? Can you tell me about your experience using Pandemic EBT cards? - Did you already use up all of the benefits on your Pandemic EBT cards? | - What has been your role in informing parents about Pandemic EBT? - What do you think is needed to improve outreach about Pandemic EBT ? |
| **Future Plans** | - Can you please tell me about your district’s plan for serving meals this fall? How prepared do you feel? What challenges do you anticipate? - Over the course of the next school year: what is your biggest concern in terms of meeting the needs of kids in your district/area? | - What are your concerns about accessing school meals in the fall? - What would make it easier to get school meals, or more likely for you to participate? | - Can you tell me how or if your organization plans to partner with the school district to provide meals this fall? |
| **Lessons learned** | - We’ve talked a lot about the challenges and the successes of your district’s meal program during the pandemic. To sum up: what is one key lesson learned that you would want to share with other food service directors? | Of all the things you have told me today, what is the one thing that you think would really help families to take advantage of the meals during this time? |  |
